# Polidocanol echosclerotherapy treatment for lymphocele: case report and review

**DOI:** 10.1590/1677-5449.190112

**Published:** 2019-12-12

**Authors:** Sergio Quilici Belczak, Gabriela Balarini Figueiredo Lima, Karin Coca Aguilar, Walter Junior Boim de Araujo, Fabiano Luiz Erzinger, Felipe Coelho

**Affiliations:** 1 Instituto de Aprimoramento e Pesquisa em Angiorradiologia e Cirurgia Endovascular – IAPACE, São Paulo, SP, Brasil.; 2 Centro Universitário São Camilo, São Paulo, SP, Brasil.; 3 Instituto Belczak de Cirurgia Vascular e Endovascular, São Paulo, SP, Brasil.; 4 Instituto da Circulação, Curitiba, PR, Brasil.; 5 Hospital Regional da Asa Norte – HRAN, Brasília, DF, Brasil.

**Keywords:** polidocanol, lymphocele, sclerotherapy

## Abstract

Formation of lymphocele secondary to transection of lymphatic channels during surgical procedures or traumas is relatively common and is reported in the postoperative period of approximately 30% of lymph node resection procedures. The condition may be asymptomatic or can present with complications such as pain, secondary infection, and compression of blood vessels, which can cause stasis, thrombosis, and edema. There is no consensus on treatment. This article describes three cases in which treatment was provided using polidocanol echosclerotherapy. Its relevance lies in the scarcity of reports in the literature.

## INTRODUCTION

Formation of lymphocele is reported in the postoperative period of approximately 30% of lymph node resection procedures. During these surgical procedures, lymphatic channels may be transected, leading to formation of abnormal collections with no epithelial lining. The interventions most often associated with this process are complete lymph node dissection (CLND) and sentinel lymph node excision (SLNE). There are also countless reports of development of lymphocele after traumas and after many other types of surgery, such as revascularization of lower extremities, inguinotomies performed to enable embolectomy or endovascular treatment of aortic aneurysms or varicose veins, and after saphenectomy for myocardial revascularization.^1^^-^ 7

In the majority of cases, lymphocele are asymptomatic and are reabsorbed, but in some situations there may be complications, including pain, secondary infection, or compression of blood vessels, causing venous stasis, thrombosis, and edema.^4^

There is not yet consensus on treatment. Many techniques have been employed, including surgical intervention, external drainage, radiotherapy, needle aspiration, and a range of sclerosing agents – povidone-iodine, bleomycin, alcohol, doxycycline, and fibrin.

Results have not been consistent, in some cases being satisfactory, in others being associated with significant side effects or elevated costs. In turn, polidocanol is widely employed for treatment of varicose veins and is a well-established sclerosing agent that chemically induces protein denaturing.^5^ This article describes use of polidocanol as a sclerosing agent for treatment of lymphocele.

## DESCRIPTION OF CASES

Three cases will be described in which patients were treated for lymphocele using polidocanol echosclerotherapy. The patients gave consent to publication of their cases and images.

### Case 1

The patient was a 34-year-old female who had been the victim of a traffic accident in which she suffered a direct trauma to the medial part of her right thigh. During the week after the accident, she developed localized pain and swelling that did not improve with analgesics and rest. Doppler ultrasonography was used to examine the venous system of her right lower limb, revealing presence of an accumulation of liquid, with no flow, in a subcutaneous region. Ultrasound-guided puncture was conducted with the objective of achieving clinical improvement of pain, withdrawing approximately 600 ml of serous yellow liquid. The patient reported absence of localized pain and improvement of the initial symptoms and there was total regression of the swelling of the thigh. She was instructed to wear 20 to 30 mmHg compression stockings. Fourteen days later, the patient returned with relapse of both swelling and pain in the medial right thigh. Puncture was performed for pain relief, combined with administration of polidocanol foam 2%, on the assumption that this was a case of lymphocele. The elastic stocking was continued and combined with local compression with a surgical compress and micropore tape, maintained for 48 h continuously. The patient recovered well, on the 14th day, wearing the elastic stocking and applying the compress daily. There was no need for analgesics and no signs of inflammation and she returned to work in 7 days, wearing the 20 to 30 mmHg elastic stocking. At 1 and 3-month follow-ups, the patient was asymptomatic, wearing the elastic stocking, but no longer applying the surgical compress (she had used it for 21 days). Control Doppler ultrasonography showed complete resolution of the liquid accumulation and the elastic stocking was withdrawn (Figures 111C). Cultures of the liquid drained were negative and its composition was compatible with lymph.

**Figure 1:**
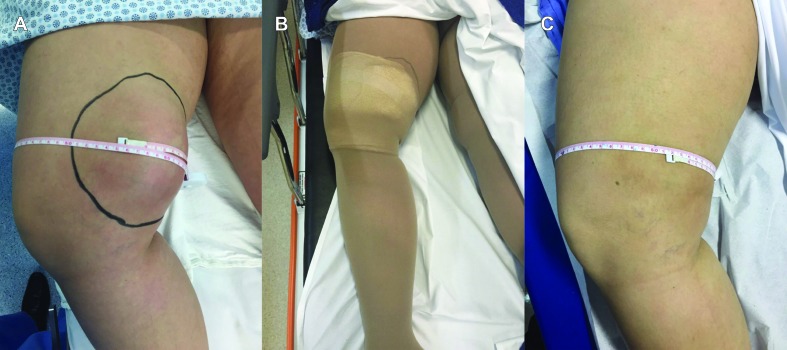
Images from Case 1: posttraumatic lymphocele. Swelling and local pain in the right medial thigh after a direct trauma (**A**). Wearing an elastic stocking over local compression dressing (surgical compress and micropore tape) after puncture to relieve pain and administer sclerotherapy with polidocanol (**B**). At follow-up appointment, patient is asymptomatic and wearing an elastic stocking. Control Doppler ultrasonography showed complete remission of accumulated liquid, and the elastic stocking was withdrawn (**C**).

### Case 2

A 46-year-old, healthy, female patient presented on the 25th day after varicose veins surgery with bilateral saphenectomy, with a mass in the mid third of the thigh that had been progressively swelling since the surgery. There were no signs of inflammation. Doppler ultrasonography revealed a liquid accumulation with no flow. She was diagnosed with lymphocele. She underwent drainage of the content (liquid with a serous appearance) of approximately 140 ml via ultrasound-guided puncture (Figure 22B) followed by injection of 10 ml of a solution of polidocanol 1% with room air at a proportion of 1:4. A compressive dressing was applied, using gauze and bandages, and a 7/8 20 to 30 mmHg elastic compression stocking was worn for 72 h continuously. The following week, the patient returned, the mass was smaller, but still present. The same process was repeated, although this time 60 ml of lymphocele content was drained. The following week the patient returned once more, with complete resolution of the condition.

**Figure 2:**
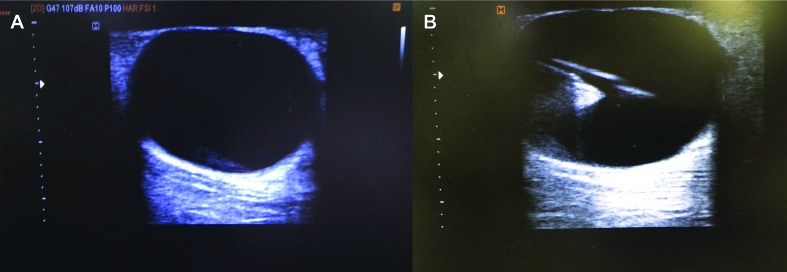
Images from Case 2: lymphocele after varicose vein surgery. Doppler ultrasonography showing liquid content with no flow, diagnosing lymphocele (**A**). Drainage of approximately 140 ml of the content (liquid, with serous appearance) by ultrasound-guided puncture, followed by injection of 10 ml of polidocanol 1% and room air at a proportion of 1:4 (**B**).

### Case 3

An 81-year-old patient suffered a fall from her own height, with considerable trauma to the area of the knee. Over the following 6 days, significant swelling developed and an ultrasonography examination was performed, revealing liquid content with no flow visible on Doppler. A diagnosis of lymphocele was made. The patient underwent drainage of the content (liquid with a serous, lemon yellow appearance), with a volume of approximately 70 ml, via ultrasound-guided puncture, followed by injection of 10 ml of a solution of polidocanol 1% and room air at a proportion of 1:4 (Figures 33B). A compressive dressing with gauze and bandages was applied and a 7/8 20 to 30 mmHg elastic compression stocking was worn for 72 h. After 2 weeks, the patient returned, with swelling reduced, but still present. The process was repeated, each time with drainage of less lymphocele content (40 and 15 ml respectively). The following week, the patient returned with the condition fully resolved.

**Figure 3:**
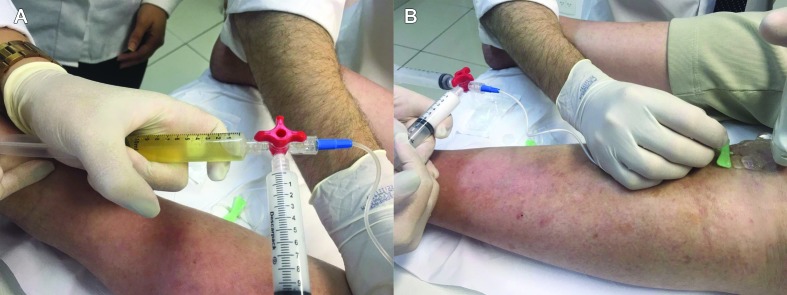
Images from Case 3: posttraumatic lymphocele. Drainage of approximately 70 ml of the lemon-yellow, serous liquid content by ultrasound-guided puncture (**A**). Injection of 10 ml of polidocanol solution 1% and room air at a proportion of 1:4 (after ultrasound-guided drainage) (**B**).

## DISCUSSION

There is scant literature on treatment of lymphocele with polidocanol. Specifically in the PubMed database, the only experience described was published by Klode in 2010. This study analyzed patients diagnosed with melanoma with a Breslow Depth exceeding 1.0 mm from January 2004 to December 2008. A total of 237 patients at stages I and II underwent SLNE and a further 53 patients underwent CLND; 33 patients developed symptomatic postoperative lymphocele, 19 after SLNE and 14 after CLND. These patients were offered surgical drainage followed by compression or ultrasound-guided sclerotherapy with polidocanol (1%). Twelve of these 33 patients agreed to the treatment using polidocanol, 8 of whom were treated successfully. In the other 4, lymphocele returned (a mean of 5.5 days after sclerotherapy), and 3 of these patients were treated successfully with a second sclerotherapy session with a higher polidocanol concentration (2%), while one patient had a second recurrence after 9 days. Considering these data, the study concluded that treatment with polidocanol (1 or 2%) is effective and safe and exhibited a higher cure rate than surgical interventions.^5^

The references of that article were also analyzed, but they were not of great relevance to the subject. On the basis of the article cited, it can be observed that lymph vessels may be transected during surgical procedures. During the postoperative period, lymphocele may form as a result of accumulation of liquid in the subcutaneous area. This is characterized as a cavity without epithelium, with fibromembranous tissue containing lymphatic fluid. Lymphatic transection can also occur after traumas or after other surgical procedures, as in the cases described in this study. Certain surgical procedures are more associated with formation of lymphocele, such as excision of sentinel lymph nodes, complete dissection of lymph nodes, prostatectomy, perineal resection to treat genitourinary cancer, bypass placement, and kidney transplant.^2^^,^ 3

It is also known that the risk of formation of lymphocele is higher in certain cases, such as: when there are scars from previous procedures, a foreign body, or the patient has comorbidities: hypertension, diabetes, and smoking.

The majority of lymphocele are asymptomatic and spontaneous resorption occurs, but from 5 to 7% of patients may exhibit postoperative complications, such as those described earlier.

There is no consensus on treatment. Surgical intervention, such as ligation of injured lymph vessels or muscle flaps, has previously been described as the first choice approach. Although success rates are relatively high, surgery involves greater risk, high morbidity, long hospital stays, and higher costs than percutaneous procedures.^2^^-^ 4

Another effective method is vacuum-assisted closure, but it is expensive. Percutaneous treatments are less invasive than surgical procedures. There have been many attempts at primary aspiration, but the methods were not effective, since 80 to 90% of lymphocele treated in this manner returned and more than 50% became infected.^8^

Many sclerosing agents have been tested, with good success rates, but some are expensive and others require long treatment times. Sclerotherapy for treatment of lymphocele during the postoperative period has been accomplished successfully using alcohol or povidone-iodine, but multiple sessions are needed with these agents to obliterate the lymph vessels; doxycycline is recommended as long as the patient is asymptomatic and drainage has already been performed, but it extends the hospital stay. Bleomycin has also been tried, less successfully, since it needs multiple sessions and has high costs and serious side effects, such as necrosis. While fibrin sealant offers excellent results in a single session, it is very expensive.^3^^,^ 9

Polidocanol is a well-known sclerosant that is widely used by vascular surgeons to treat varicose veins. Efficacy of foam at 1% to 2% is superior to the liquid form of this agent, since it induces an inflammatory reaction in the fibromembranous wall of the lymphocele, in addition to reaching the injured lymphatic ducts, provoking adhesion and fibrosis.^10^

It was observed that combining compression with sclerotherapy with polidocanol reduced the duration of treatment, increased fluid drainage, and reduced the risk of complications, such as infection, discomfort, and other side effects, when compared with compression alone.

Therefore, use of polidocanol foam at 1 or 2% to treat lymphocele is a new method that is effective, simple, and safe and has a good cost-benefit profile. The rate of cure is better than observed with surgical interventions and no complications such as infection at the procedure site were observed and there was no need for surgery.
