# Supplementary material for: Polidocanol echosclerotherapy treatment for lymphocele: case report and review
Source: J Vasc Bras. 2019 Dec 12;19:e20190112. doi: 10.1590/1677-5449.190112 (PMC6944319; doi:10.1590/1677-5449.190112)
Supplement: Translation

# Translation: Tratamento de linfocele mediante ecoescleroterapia com polidocanol: relato de casos e revisão

**Authors:** Sergio Quilici Belczak, Gabriela Balarini Figueiredo Lima, Karin Coca Aguilar, Walter Junior Boim de Araujo, Fabiano Luiz Erzinger, Felipe Coelho

**Affiliations:** 1 Instituto de Aprimoramento e Pesquisa em Angiorradiologia e Cirurgia Endovascular – IAPACE, São Paulo, SP, Brasil.; 2 Centro Universitário São Camilo, São Paulo, SP, Brasil.; 3 Instituto Belczak de Cirurgia Vascular e Endovascular, São Paulo, SP, Brasil.; 4 Instituto da Circulação, Curitiba, PR, Brasil.; 5 Hospital Regional da Asa Norte – HRAN, Brasília, DF, Brasil.

**Keywords:** polidocanol, linfocele, escleroterapia

## Abstract

A formação de linfocele, resultante da transecção dos canais linfáticos durante procedimentos cirúrgicos ou traumas, é relativamente comum, sendo relatada em aproximadamente 30% dos pós-operatórios de ressecção de linfonodos. Ela pode evoluir assintomática ou pode apresentar complicações, como dor, infecção secundária, compressão de vasos sanguíneos, a qual evolui para estase, trombose e edema. Seu tratamento ainda é de difícil consenso. Este artigo propõe descrever três casos em que o tratamento foi realizado a partir de ecoescleroterapia com polidocanol. Sua relevância está na escassez de relatos na literatura.

## INTRODUÇÃO

A formação de linfocele é reportada em aproximadamente 30% dos pós-operatórios de ressecção de linfonodos. Durante o procedimento cirúrgico, pode ocorrer transecção dos canais linfáticos, formando coleções anormais sem uma cápsula epitelial de revestimento. As intervenções mais associadas a esse processo são a dissecção completa de linfonodos (CLND) e a retirada de linfonodo sentinela (SLNE). Há também inúmeros relatos do desenvolvimento de linfocele pós-trauma e em diversas outras cirurgias, como revascularização dos membros inferiores, inguinotomias para realização de embolectomia ou tratamento endovascular de aneurismas aórticos, de varizes e após safenectomia para revascularização miocárdica^1^^-^ 7.

Na maioria dos casos, a linfocele é assintomática e reabsorvida, entretanto, em algumas situações, pode apresentar complicações, repercutindo com dor, infecção secundária, compressão de vasos sanguíneos resultando em estase venosa, trombose e edema^4^.

O tratamento é de difícil consenso. Várias técnicas foram realizadas, como intervenção cirúrgica, drenagem externa, radioterapia, aspiração por agulha e vários agentes esclerosantes – povidona iodo, bleomicina, álcool, doxiciclina e fibrina.

Os resultados não foram consistentes, sendo ora satisfatórios, ora apresentando efeitos colaterais significativos ou alto custo. Por sua vez, o polidocanol, muito usado no tratamento de varizes, é um agente esclerosante bem estabelecido, sendo um indutor químico que resulta na desnaturação proteica^5^. Neste relato, será descrito o uso de polidocanol como agente esclerosante no tratamento de linfocele.

## DESCRIÇÃO DOS CASOS

Serão relatados três casos de pacientes tratadas de linfocele mediante ecoescleroterapia com polidocanol. Os pacientes consentiram ter seus casos relatados e suas imagens divulgadas.

### Caso 1

Paciente feminina, 34 anos, sofreu acidente automobilístico, com trauma direito em coxa medial direita; evoluiu na semana seguinte, com dor local e aumento de volume sem melhora com analgésico e repouso. Foi submetida a exame de eco-Doppler venoso do membro inferior direito, o qual revelou a presença de coleção líquida sem fluxo em região subcutânea. Optou-se por realizar punção ecoguiada para melhora clínica da dor, com saída de aproximadamente 600 ml de líquido amarelado seroso. A paciente evoluiu sem dor local e com melhora dos sintomas iniciais, assim como regressão total do aumento de volume na coxa, mantida meia elástica 20 a 30 mmHg. Após 14 dias, a paciente retornou com recorrência do aumento de volume e da dor na coxa medial direita; optou-se por punção de alívio associada à realização de 10 ml de espuma com polidocanol a 2%, pensando no caso de linfocele, seguido do uso da meia elástica, associado a uma compressão local utilizando compressa cirúrgica envolvida com micropore por 48 h contínuas. A paciente evoluiu favoravelmente, no 14º dia, utilizando diariamente a meia elástica e a compressa. Não houve necessidade do uso de analgésicos, sem a presença de sinais flogísticos e com retorno a sua atividade laboral em 7 dias, utilizando meia elástica 20 a 30 mmHg. No retorno de 1 e 3 meses, a paciente assintomática em uso de meia elástica, não mais usando a compressa cirúrgica (usou por 21 dias); eco-Doppler de controle, evidenciando remissão completa da coleção líquida, sendo suspenso o uso da meia elástica (Figura 111C). A cultura do líquido drenado veio negativa e com composição compatível com linfa.

**Figura 1:** Imagens do Caso 1: linfocele pós-trauma. Aumento de volume e dor local em coxa medial direita após trauma direto (**A**). Uso de meia elástica e compressão local (compressa cirúrgica e micropore) após punção de alívio associada à realização de escleroterapia com polidocanol (**B**). Em retorno, paciente assintomática e em uso de meia elástica, com eco-Doppler de controle evidenciando remissão completa da coleção líquida, sendo por sua vez suspenso o uso da meia elástica (**C**).

### Caso 2

Paciente de 46 anos, sexo feminino, hígida, no 25º dia de pós-operatório de cirurgia de varizes com safenectomia bilateral evoluiu com tumoração, sem sinais flogísticos, de crescimento progressivo em terço médio da coxa desde a cirurgia, realizou ultrassonografia Doppler evidenciando conteúdo líquido sem fluxo ao Doppler. Diagnosticou-se linfocele. A paciente foi então submetida à drenagem do conteúdo (de aspecto seroso e líquido), de aproximadamente 140 ml mediante a realização de punção ecoguiada (Figura 22B) e, então, realizou-se a injeção de 10 ml da solução polidocanol 1% e ar ambiente na proporção 1:4. Realizou-se curativo compressivo com gazes e atadura e colocação de meia elástica compressiva 7/8 20 a 30 mmHg por 72 h contínuas. Na semana subsequente, a paciente retornou com a tumoração de tamanho reduzido, mas ainda presente. Realizou-se novamente o processo, porém, dessa vez, com drenagem de 60 ml do conteúdo da linfocele. Na semana subsequente, a paciente retornou com resolução completa do quadro.

**Figura 2:** Imagens do Caso 2: linfocele pós-cirurgia de varizes. Ultrassonografia Doppler evidenciando conteúdo líquido sem fluxo ao Doppler, diagnosticando linfocele (**A**). Drenagem do conteúdo (de aspecto seroso e líquido), de aproximadamente 140 ml mediante a realização de punção ecoguiada, seguida de injeção de 10 ml de polidocanol a 1% e ar ambiente na proporção 1:4 (**B**).

### Caso 3

Paciente de 81 anos apresentou histórico de queda da própria altura com trauma importante na região genicular. Evoluiu em 6 dias, com abaulamento importante na região e realizou exame de ultrassonografia que evidenciou conteúdo líquido sem fluxo ao Doppler. O diagnóstico foi linfocele. A paciente foi submetida à drenagem do conteúdo (de aspecto seroso amarelo citrino e líquido), de aproximadamente 70 ml mediante a realização de punção ecoguiada e, então, realizou-se a injeção de 10 ml da solução polidocanol 1% e ar ambiente na proporção 1:4 (Figura 33B). Realizou-se ainda curativo compressivo com gazes e atadura e colocação de meia elástica compressiva 7/8 20 a 30 mmHg por um período de 72 h. Após 2 semanas, a paciente retornou com a tumoração de tamanho reduzido, mas ainda presente. Realizou-se novamente o processo, porém cada vez com drenagem de menor conteúdo de linfocele (40 e 15 ml respectivamente). Na semana subsequente, a paciente retornou com resolução completa do quadro.

**Figura 3:** Imagens do Caso 3: linfocele pós-trauma. Drenagem do conteúdo, apresentando aspecto seroso amarelo citrino e líquido, de aproximadamente 70 ml mediante a realização de punção ecoguiada (**A**). Injeção de 10 ml da solução polidocanol a 1% e ar ambiente na proporção 1:4 (posterior à drenagem ecoguiada) (**B**).

## DISCUSSÃO

Há escassez de literatura referente ao tratamento de linfocele com polidocanol. Especificamente, na base de dados PubMed, a única experiência relatada foi por Klode em 2010. Nesse estudo, foram analisados pacientes diagnosticados com melanoma, com Índice de Breslow acima de 1,0 mm, no período de janeiro de 2004 a dezembro de 2008. Um total de 237 pacientes, em estágio I e II, receberam SLNE, enquanto 53 receberam CLND; 33 pacientes desenvolveram linfocele pós-operatória sintomática, sendo 19 após SLNE e 14 após CLND. Foi proposta a esses pacientes a drenagem cirúrgica seguida de compressão ou escleroterapia com polidocanol (1%) guiada por ultrassom. Dos 33 pacientes, 12 concordaram com o tratamento à base de polidocanol, sendo 8 tratados com sucesso e 4 apresentando recorrência (em média de 5,5 dias após a escleroterapia), entre eles, 3 foram tratados com sucesso após nova escleroterapia com concentração maior de polidocanol (2%) e um paciente desenvolveu uma segunda recorrência após 9 dias. Considerando esses dados, o estudo concluiu que o tratamento com polidocanol (1 ou 2%) é efetivo e seguro, além de apresentar índice de cura maior do que intervenções cirúrgicas^5^.

Foram pesquisadas também as referências do artigo incluído, sem grande relevância para o tema abordado. Com base no artigo citado, observa-se que, durante procedimentos cirúrgicos, vasos linfáticos podem ser transeccionados. No pós-operatório, pode haver a formação de linfocele, que resulta do acúmulo de líquido no subcutâneo. Sendo caracterizada por uma cavidade não epitelizada com tecido fibromembranoso contendo fluido linfático. Essa transecção linfática também pode ocorrer pós-trauma e outros procedimentos cirúrgicos, como nos casos relatados neste estudo. Há procedimentos cirúrgicos que estão mais associados à formação de linfocele, como a retirada de linfonodo sentinela, dissecção completa de linfonodo, prostatectomia, resseccção perineal devido a câncer geniturinário, passagem de *bypass* e transplante renal^2^^,^ 3.

Sabe-se também que o risco de formação de linfocele aumenta em alguns casos, como: presença de cicatrizes de procedimentos anteriores ou corpo estranho e comorbidades do paciente: hipertensão, diabetes e uso de tabaco.

A maioria das linfoceles é assintomática e com reabsorção espontânea, porém de 5 a 7% dos pacientes em pós-operatório podem apresentar complicações, como as descritas anteriormente.

Não existe consenso sobre o tratamento. A intervenção cirúrgica, como ligação dos vasos linfáticos lesados ou retalho muscular, já foi descrita como o padrão de escolha. Apesar da taxa de sucesso relativamente alta, a cirurgia acarreta maior risco, alta morbidade, hospitalizações longas e custo maior do que o procedimento percutâneo^2^^-^ 4.

Outro método eficiente é a terapia de fechamento a vácuo, porém apresenta alto custo. As terapias percutâneas são menos invasivas do que procedimentos cirúrgicos; diversas tentativas de aspiração primária foram feitas, porém não eram métodos efetivos, uma vez que 80 a 90% das linfoceles tratadas desse modo recorreram e mais de 50% infectaram^8^.

Diversos agentes esclerosantes foram testados, com boa taxa de sucesso, porém alguns envolviam alto custo e outros demandavam muito tempo de terapia. A escleroterapia no tratamento de linfocele no pós-operatório foi realizada com sucesso empregando álcool ou povidona-iodada, mas esses agentes requeriam múltiplas sessões para obliterar os vasos linfáticos; doxiciclina é recomendada desde que o paciente esteja assintomático e a drenagem já tenha sido realizada, porém, prolonga o tempo de hospitalização. A bleomicina também foi tentada com menos sucesso, uma vez que requeria múltiplas sessões, alto custo e efeitos colaterais graves, como necrose. O uso de selante de fibrina, embora apresente excelentes resultados em uma única sessão, tem um custo muito alto^3^^,^ 9.

O polidocanol é um esclerosante bem conhecido e muito usado no tratamento de varizes por cirurgiões vasculares. A eficiência da espuma a 1% a 2% foi superior à da forma líquida desse agente, uma vez que induz uma reação inflamatória da parede fibromembranosa da linfocele, além de atingir os ductos linfáticos lesados produzindo adesão e fibrose^10^.

Foi visto que, ao associar compressão e escleroterapia com polidocanol, o tratamento tem menor duração, a drenagem de fluido é maior e o risco de complicações, como infecção, desconforto e outros efeitos colaterais, é menor do que quando comparado só à compressão.

Portanto, o uso de polidocanol em espuma a 1 ou 2% para tratar linfocele é um método novo, efetivo, fácil, seguro e com boa relação entre custo e benefício. A taxa de cura é melhor do que quando comparada a de intervenções cirúrgicas, não sendo vistas complicações, como infecção do local do procedimento, nem necessidade de abordagem cirúrgica.
